# The use of cognitive psychology-based human-computer interaction tax system in ceramic industry tax collection and management and economic development of Jingdezhen city

**DOI:** 10.3389/fpsyg.2022.944924

**Published:** 2022-10-20

**Authors:** Mingqing Jiao

**Affiliations:** ^1^School of Management and Economics, Jingdezhen Ceramic University, Jingdezhen, China; ^2^School of Management and Economics, Beijing Institute of Technology, Beijing, China

**Keywords:** HCI, cognitive psychology, ceramic industry, tax collection and management, industrial economic development, Jingdezhen

## Abstract

This work aims to solve the complex problems of non-linearity, instability, and multiple economic factors in the tax forecast of the ceramic industry to ensure the sustainable development of the ceramic industry. The key influential indicators of the tax forecast are obtained by analyzing the principal components affecting the tax index. In addition, a human-computer interaction (HCI) system is established based on cognitive psychology theory to improve the user-friendliness of tax analysis. At the same time, the tax data of the ceramic industry in Jingdezhen City, Jiangxi Province in different years are used for the empirical analysis of the tax prediction of different prediction models, including particle swarm optimization (PSO) algorithm, and fusion algorithm (FA), and support vector machine (SVM). This work comprehensively analyzes the influence of the optimized tax supervision mode on the economic development of the ceramic industry and provides ideas for the development of the ceramic industry in Jingdezhen. The research results demonstrate that the main indicator affecting tax revenue is the added value of the primary and secondary industries. The optimized SVM based on grid search method can provide a comprehensive data base for tax forecasting. The optimization of the computer system based on cognitive psychology improves the model prediction accuracy by 10%, and the absolute error decreases from 6.9 to 1.8%. The tax forecast results indicate that the tax imbalance in Jingdezhen is increasing. Therefore, the government needs to attach great importance to the development of the ceramic industry and strictly implement the tax policy. The tax supervision model can alleviate the problems of low fiscal contribution rate, tax evasion, and management loopholes. In addition, the SVM tax prediction model optimized by grid search method will lay a theoretical foundation for the research and application of taxation in the ceramic industry.

## Introduction

The rapid progress of China’s economy, the deepening of domestic reform, and the accelerated pace of opening up have sped up China’s economic development (Wang et al., 2016; Zheng and Walsh, 2019; Bruton et al., 2021). Taxation is the main source of China’s fiscal revenue and affects the per capita disposable income and residents’ quality of life. It is also closely related to enterprises’ financial burden and development (Li et al., 2019; Xiao, 2020). Taxation helps the government reallocate resources, adjust economic structure, promote employment, and stabilize people’s life. It provides a basic guarantee for the rapid and stable development of the national economy (Gilley, 2017; Fan and Liu, 2020). Tax forecast considers the changes of factors affecting tax revenue and historical tax data and implements scientific judgment on the prospect of tax revenue through specific models and analysis methods (Graham et al., 2017). On the one hand, it is necessary to predict and analyze the tax revenue before the change of national tax policy. The prediction results can help the tax personnel arrange the tax plan and tax situation effectively, providing a theoretical basis for the decision-making of relevant departments (Bratten et al., 2017). On the other hand, a proper tax platform can adjust resource allocation, expand the scale of related enterprises, and achieve sustainable development, taking into account some industry development and income imbalances.

Human-computer interaction (HCI) is a discipline for designing and evaluate computer systems for human use. Specifically, HCI improves the efficiency of people using computers by improving the input and output of computers. Computer science has had a profound influence on the development of cognitive psychology, studying the use of new computer interface methods to optimize the design of desired attributes (learning ability, probing ability, and use efficiency). From the perspective of cognitive psychology (Choi and Luo, 2019), HCI combines computer interfaces with psychological patterns of human activity and computer interfaces with existing social practices or existing sociocultural values. It can provide a practical communication point for analyzing socio-economic and cultural development and establish a circulating information exchange network between people, computers, and society.

Jingdezhen city in Jiangxi Province is famous for its ceramics. In Jingdezhen, there are about 3,000 ceramic enterprises, 96% of which are private enterprises. A series of problems, such as the management system of small workshops and the large tax burden of large and medium-sized ceramic enterprises, have hindered the development of the ceramic industry (Mirfakhradini et al., 2018; Atilgan Türkmen et al., 2021). In recent years, due to the high attention of the Jiangxi provincial Party committee and government, the tax department has continuously strengthened its tax work. However, the current ceramic tax collection and management situation is not optimistic because of the complexity of the industry. The main problems are that the industrial development is relatively backward, the protection of technology inheritance is not high, and the lack of cooperation opportunities with ceramic research institutions. Therefore, using new techniques and methods to achieve accurate tax forecasting is of great significance for solving problems in the ceramic industry.

Research on the precise tax forecast mainly focuses on applying new algorithms to build diverse forecast models. Höglund (2017) used a genetic algorithm (GA) combined with a linear discriminant analysis model. The analysis results of the tax data of many enterprises proved that the model can effectively judge whether the inspected enterprises pay taxes normally. Mabe-Madisa (2018) used practical learning classification intelligent algorithms to classify compliant and non-compliant taxpayers. Rahimikia et al. (2017) utilized a hybrid intelligent system consisting of the multi-layered perceptron neural network, the support vector machine (SVM), logistic regression classification model, and harmonious search optimization algorithm to detect tax evasion by Iranian enterprises. The authors verified the model’s effectiveness in taxation. Namazi and Sadeghzadeh Maharluie (2018) applied the decision tree algorithm to forecast the tax evasion of listed companies in the Tehran Stock Exchange (TSE). They found that the algorithm could effectively supervise the tax evasion of TSE listed companies. Andini et al. (2018) effectively supervised taxation and provided information for tax policy decisions by using machine learning algorithms. Lee (2021) researched the accuracy of economic tax expense forecasts and examined whether the strength of investor protection affects analysts’ tax expense forecast accuracy. The findings suggested that firms with high accuracy in analysts’ tax forecasts have low tax avoidance levels. Dobroviè et al. (2021) established an analysis and prediction model of tax evasion in European Union (EU) countries. Through the econometric prediction model, EU countries were divided into five reference groups according to the value of value-added tax (VAT) difference according to the results of cluster analysis. The results showed that taking appropriate measures can improve the accuracy of forecasts in the economic field. The above studies show that the use of new algorithms and models for tax forecasting has become one of the research hotspots in this field.

In the research results of the above-mentioned stages, the data of tax indicators are mainly carried out through principal component analysis (PCA) to eliminate redundant variables between tax indicators. In addition, SVM, particle swarm optimization (PSO) algorithm, and fusion algorithm (FA) are used to conduct in-depth training on the tax data of different years of the ceramic industry in Jingdezhen City, Jiangxi Province. A HCI system is established based on cognitive psychology to improve the analysis efficiency. Finally, the different models are compared and analyzed to determine the effectiveness of the appropriate model. In addition, a reasonable solution to the tax problem in Jingdezhen City is proposed based on the influence of the tax supervision mode on the economic development of the ceramic industry. The innovation of the research lies in the comparison of different algorithms, providing a creative study for tax administration. This work offers a theoretical basis for the sustainable development of the ceramic industry in Jingdezhen City, Jiangxi Province.

## Materials and methods

### Current status of tax collection and management of the ceramic industry

The current status of ceramic industry development in Jingdezhen is understood by reviewing the literature. The current problems in taxation are as follows. First, the macroscopic tax burden of the ceramic industry is low, and the tax share of the ceramic industry is far lower than the total output value. In recent years, the tax burden of the ceramic industry in Jingdezhen only accounts for 1.3% of the macroscopic industry tax burden. 2013 witnessed the highest proportion, which was 1.4%. In 2015, the proportion was only 0.74%. The data show that the ceramic industry, as the primary source of income in Jingdezhen, has not been reflected in taxation. Second, the types of enterprises are diversified, but there are few large and medium-sized enterprises. There were 6,525 ceramic enterprises and 4,261 individual enterprises from 2014 to 2018. In 2016, only 10 enterprises had a tax payment of more than 10 million Chinese Yuan (CNY), and only three enterprises had a tax payment of 500,000 to 1 million CNY. Therefore, most of the enterprises in Jingdezhen are small enterprises, which severely restricts the expansion of the national tax revenue. Third, tax payment is principally based on value-added tax, corporate income tax, and personal income tax. There are a few types of taxes, and the output value of each tax is low.

The main problems in the development of Jingdezhen ceramic industry are as follows. First of all, the ceramic industry chain is long, and there are drawbacks in the management of the tax management department. Due to the wide range of material sources in the ceramic industry, the chains between various industries are fragmented, and there is no suitable sharing platform. As a result, it is challenging for tax officials to grasp key tax indicators such as the utilization rate of raw materials and electricity consumption. Second, tax evasion prevails. The proportion of ceramic tax continues to decline with the continuous development of the ceramic industry. For example, the tax administration did not report ceramic artists truthfully; nonetheless, most studios and small businesses did not enforce tax policies, which contributed to a climate of tax evasion. Third, the tax collection and management of ceramics is weak. While the number of tax bureaus continues to increase every year, most are senior executives, and the number of ceramic tax collectors has not increased, making tax collection incredibly difficult. Fourth, the sharing of information on tax collection and management of ceramics is low. The ceramic industry takes its information protection very seriously. Therefore, it is difficult for the tax management department to obtain relevant information of taxpayers and to ensure the authenticity of the obtained information, resulting in the problem of information asymmetry.

### Human-computer interaction model of support vector regression combined with cognitive psychology

Cognitive psychology refers to a process of thinking and reasoning based on mental processing (Biggs et al., 2018). The changing process of cognitive psychology is a complex psychological mechanism, primarily including feeling, perception, memory, thinking, and language. When the human brain receives external information, it needs to process the input information and convert it into internal psychological activities to control human behavior. From the perspective of cognitive psychology, the HCI process is a person’s cognitive process. The improvement of humanistic characteristics in human-computer interface is to improve the efficiency and availability of information interaction in human-computer interface, prevent cognitive errors, and improve the cognitive efficiency of human beings. Based on the analysis of the user’s thinking and behavior model, a comprehensive cognitive model is established through the analysis of the human thinking process to find out the main factors affecting the efficiency of HCI. It is necessary to encode the external stimulus information in a certain way under HCI so that the brain can use the information. The encoded information becomes a mental representation, representing the process by which the computer or human brain processes the model data. It eventually stays in the human consciousness and is long-term stored in the working memory for future use in human life and work.

**FIGURE 1:**
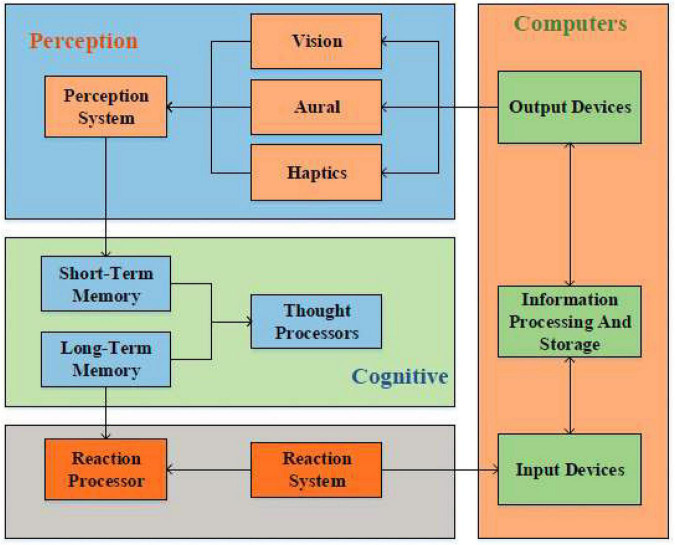
Information processing process of the human-computer interaction (HCI) system.

The human-machine interface becomes the communication point between the human user and the computer. In addition, the HCI subjects include computers and computer users. Information processing models are used to process received stimulus information and take corresponding actions through perception, cognition, and response systems (Renoult and Mendelson, 2019). HCI is the intersection of computer science technology and cognitive psychology. In other words, HCI uses knowledge from cognitive psychology to help humans process relevant external information. The HCI system is designed based on cognitive psychology theory. Figure 1 shows the information processing process of the HCI system.

**FIGURE 2:**
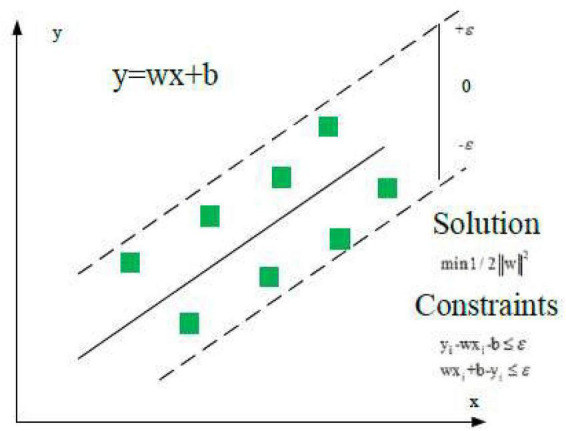
Schematic diagram of support vector regression (SVR) model.

Support vector regression is a binary classification model and a general term for the supervised learning model and its related learning algorithms. It is used for classification analysis and regression analysis of data. Its basic model is a linear classifier with intervals defined in the feature space. The *x* input space is mapped to a high-level feature space through nonlinear transformation, and the linear function of this feature space is used to fit the sample data. Figure 2 is its schematic diagram. In the feature space, the linear function is defined as Eq. (1).


(1) $$y=\text{f}\,\left(x\right)=w^{T}\,\varphi \,\left(x_{1}\right)+b$$

In Eq. (2), *w* denotes the weight; *b* is the offset top; φ(*x*_1_) represents the nonlinear mapping function; s. t. y1 means making y1 satisfy a certain equation; α denotes the Lagrange multiplier vector. Then, the function can be written as:


(2) $$\text{Min}\,J_{1}\,\left(w,b,e\right)=1/2\,{∥w∥}^{2}+1/2\,C\,\sum_{i=1}^{N}e_{i}^{2}$$

(3) $$s.t.y_{1}=w\,T\,\varphi \,\left(x_{i}\right)+b+e_{i},i=1,2,N$$

This work introduces the Lagrangian multiplier, forming the Lagrangian function:


(4) $$L_{i}\,\left(w,b,e,a\right)=J_{i}\,\left(w,b,e\right)+\sum_{\text{i}=1}^{N}\alpha_{i}\,\left(y_{i}-{\text{w}}^{T}\,\varphi \,\left(x_{i}\right)-\text{b}-{\text{e}}_{i}\right)$$

There are Eq. (5) ∼ Eq. (8) according to the Karush–Kuhn–Tucker condition.


(5) $$\frac{\partial L_{1}}{\partial w}=0\Rightarrow \text{w}=\sum_{\text{i}=1}^{N}\alpha_{i}\,\varphi \,\left(x_{i}\right)$$

(6) $$\frac{\partial L_{1}}{\partial w}=0\Rightarrow \sum_{\text{i}=1}^{N}\alpha_{i}=0$$

(7) $$\frac{\partial L_{1}}{\partial w}=0\Rightarrow e_{i}=\frac{1}{C}\,\alpha_{i},\text{i}=1,2\,N$$

(8) $$\frac{\partial L_{1}}{\partial w}=0\Rightarrow y_{i}={\text{w}}^{T}\,\varphi \,\left(x_{i}\right)+\text{b}+{\text{e}}_{i},\text{i}=1,2\,N$$

In the feature spaces φ(*x*_*i*_) and φ(*x*_*j*_), the product of φ(*x*_*i*_)^*T*^ and φ(*x*_*j*_) needs to satisfy the Mercer condition that can be used as the kernel function. *k*(*x*_*i*_,*x*_*j*_) = φ(*x*_*i*_)^*T*^φ(*x*_*j*_), where *k*(*x*_*i*_,*x*_*j*_)is the kernel function. Here, a Gaussian radial basis kernel function with good generalization ability is utilized, which can be expressed as Eq. (9).


(9) k⁢(xi,xj)=exp-xi-xj2/δ2

Eq. (10) describes the SVR matrix.


(10) [A⁢EET⁢  0]⁢[αb]=[y0]

In Eq. (10), *A* = *K* + *V*,*K* = (*k*_*ij*_)_*N*×*N*_^,^ *V* = diag(1/*C*,1/*C*1/*C*); E stands for an Nx1 matrix with all elements being 1. Therefore, the regression model of SVR can be written as Eq. (11).


(11) $$f\,\left(x\right)=\sum_{\text{i}=1}^{N}\alpha_{i}\,k\,\left(x,x_{i}\right)+b$$

### Forecast algorithms of taxation data

**FIGURE 3:**
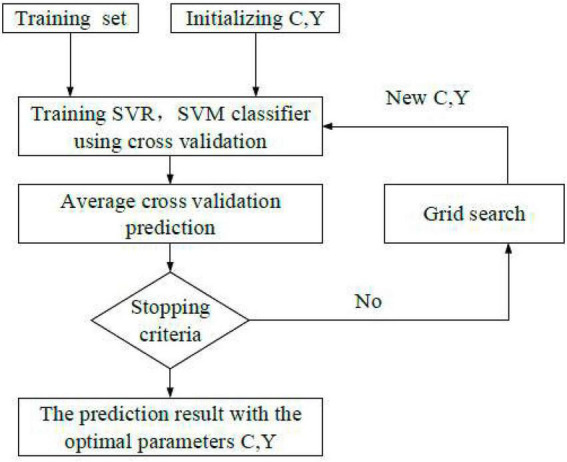
Algorithm flow of GS.

**FIGURE 4:**
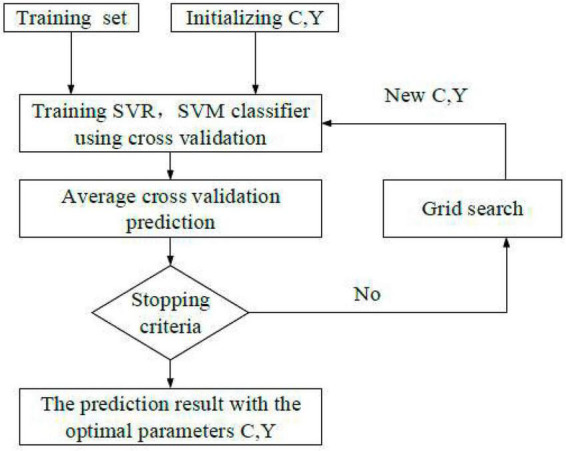
Algorithm flow of GM (1,1).

(1) The gale-shapley (GS) algorithm is a new algorithm based on cross-validation (CV). Figure 3 presents the K-CV method used here. First, the original data are averagely divided into K combinations. Data corresponding to each set is a test unit, and the remaining K-1 sets are training sets. Then, K models are obtained after training, and the accuracy of each model after all classifications is averaged and utilized as the performance evaluation indicator of each model. The parameters are optimized by selecting a gridded component in a certain area, and the combination with the highest accuracy among all the combinations is searched and regarded as the optimal parameter combination of parameters.

(2) Grey models (GM) (1,1) generates a set of new data series with apparent trends for some data series accumulatively. Besides, a model to make forecasts is built according to the growth trend of the new data series. Then, the reverse calculation is performed by the subtraction method to restore the original data sequence, thereby obtaining the prediction results, as shown in Figure 4 (Han and Hong, 2018).

(3) Genetic algorithm shows the process of natural selection based on the theory of biological genetics. In this process, the most suitable individuals are selected for reproduction. The population evolved through replication, crossover, mutation, and other operations, according to the theories about the survival of the fittest and information exchange (Metawa et al., 2017). The optimization process of GA is as follows. First, the genetic population is decoded into the SVM model, and the fitness is calculated after substituting the individuals into the model. If the conditions are not met, the individuals will return to the genetic population for selection. The individuals can pass the optimized SVM model if the conditions are met.

(4) The PSO algorithm is inspired by bird swarms searching for something, which arranges complex events systematically and logically. It can provide candidate solutions (Wang et al., 2018). The essence of the fundamental particle swarm algorithm’s continuous optimization algorithm is iterative update optimization, which can randomly assign and select multiple particles. Each substance’s gradual adaptation value can be calculated according to the adaptation function’s definition. In the update of the intermediate position velocity, the optimal position can be changed according to the above equations, and the gradual adaptation value can be repeatedly calculated. When the search for the global optimal combined solution ends, the particles in the algorithm update the original position and velocity according to the velocity position. The particle velocity update equation in dimensional space can be written as:


(12) $$V_{i\,q}=V_{i\,q}+C_{1}\,\text{rand}\,\left(\right)\,\left(p_{\text{iq}-}\,X_{\text{iq}}\right)+C_{2}\,\text{rand}\,?\,\left(\right)\,\left(p_{\text{gq}}-{\text{x}}_{\text{iq}}\right)$$

(13) $$\left\{ \begin{array}{c} V_{\text{iq}}={\text{V}}_{m\,a\,x},\mathrm{if}\,{\text{V}}_{\text{iq}}>{\text{V}}_{m\,a\,x}\\ V_{\text{iq}}=-{\text{V}}_{m\,a\,x},\mathrm{if}\,{\text{V}}_{\text{iq}}<-{\text{V}}_{m\,a\,x} \end{array}\right.$$

where *C*1 stands for the historical optimal weight coefficient when the particle itself searches; *C*2 represents the global optimal weight coefficient when the particle itself searches; rand1 () and rand2 () refer to random numbers between 0–1; *Vmax* denotes the maximum velocity limit; *Viq* signifies the velocity of some spatial dimension. Eq. (14) indicates the particle position update in q-dimensional space.


(14) $$X_{i\,q}=X_{i\,q}+r\,V_{i\,q}$$

In Eq. (14), *r* refers to the elastic coefficient of the velocity variable in the update equation. The original historical position and velocity of the particle and the state and position of the historical movement of the population affect the velocity and position of the particle in the population. The goal is to find the optimal combination through complex cooperation.

### Principal component analysis

Principal component analysis is a linear statistical method in feature extraction to reduce the dimension of input features from the original data (Aït-Sahalia and Xiu, 2019). Orthogonal transformation is utilized to convert the set of *n* samples of P possible related features into *n* sample sets of *m (m < 1)* unrelated features of the principal component. First, the random variables are collected; the n samples form a sample array, and the following transformation is made to the sample array:


(15) Zij=xij-xj-sj,i=1,2n;;j=1,2p

Eq. (15) is standardized to obtain the coefficient matrix associated with the standardized matrix *Z*.


(16) $$R={\left(r_{i_{j}}\right)}_{p\times p}=\frac{Z^{T}\,Z}{\text{n}-1}$$

The characteristic equation of the matrix related to the sample is substituted to find the characteristic root of p and determine the *m* value according to the cumulative rate of the eigenvalues. Finally, the orthogonalized variables are converted into principal components to comprehensively evaluate the *m* principal components. In addition, the final evaluation value is solved through weighted summation. The weight is the variance contribution rate of each principal component.

### Normalization processing

Many scholars have verified that experimental data of different dimensions will affect the forecast results and model accuracy of the forecast model (Tian et al., 2017). The measurement units of the included taxation data are different, and the difference in the data value of related indicators is also large. It is necessary to standardize the taxation data before model training, i.e., to normalize all data to the [0, 1] interval. This operation can eliminate the impacts of the difference between forecast indicator data on the performance of the taxation forecast model. The specific processing is shown in Eq. (17).


(17) xi-i=xi-xm⁢i⁢nxm⁢i⁢n⁢nm⁢a⁢x

In Eq. (17), xi- represents the normalized taxation value, *x_i_* represents the index column data, and*x*_*max*_,*x*_*min*_ represents the maximum and minimum values of the data in the index column, respectively.

### Model evaluation indicators

Three evaluation indicators are designed as follows. (1) Mean absolute error (MAE) is the average of the absolute values of the deviations of all individual observations from the arithmetic mean. MAE can avoid the problem that the errors cancel each other out. Therefore, it can accurately show the actual forecast error. (2) Mean square error (MSE) is a measure that indicates the degree of difference between the quantity to be estimated and the estimating quantity. (3) Root mean square error (RMSE) is the square root of the ratio of the square of the deviation of the forecast value and the true value to the number of observations n. In the actual measurement, the number of observations n is always limited, and the true value can only be indicated by a reliable (optimal) value instead. In Eq. (18) ∼ Eq. (20), represents the actual value, and vi represents the forecast value.


(18) $$M\,A\,E\,\frac{1}{n}\,\sum_{i=1}^{n}\left|\hat{y\,l}-y\,i\right|$$

(19) $$M\,S\,E=\frac{1}{n}\,\sum_{i=1}^{n}{\left|\hat{y\,l}-y\,i\right|}^{2}$$

(20) $$R\,M\,S\,E=\sqrt{\frac{1}{n}\,\sum_{i=1}^{n}{\left|\hat{y\,l}-y\,i\right|}^{2}}$$

### Sample data collection and analysis

The total tax revenue data (Y, ten thousand CNY) of Jingdezhen City from 2009 to 2019 are chosen. The following ten major impact indicators are through literature review, according to the size of the influencing factors. The added value of the primary, secondary, and tertiary industries is X1, X2, and X3, respectively; the total industrial output value is X4; the fixed asset investment is X5; the total retail sales of social consumer goods is X6; the commodity sales is X7; the total value of foreign trade import and export is X8; the deposit balance of urban and rural residents is X9. The measuring unit of all indicators is 10,000 CNY. The experimental data come from *China Statistical Yearbook* and *China Tax Yearbook*. The data from 2000 to 2010 are selected as the training data, and the data from 2015 to 2019 are selected as the test data. Table 1 summarizes the detailed data.

**TABLE 1:** Tax revenue model data.

Years	Y	X_1_	X_2_	X_3_	X_4_	X_5_	X_6_	X_7_	X_8_	X_9_
2019	554.3833	685.8533	3,646.61	3,920.037	27,259.62	7,479.56	2,807.2	750.253	1,697,5870	4,005.6
2018	505.0033	625.7767	3,360.387	3,586.007	2,684.36	7,358.25	2,522.133	700.4	16,062,528	3,956.8
2017	490.3667	611.7533	3,209.327	2,847.69	2,596.53	7,361.78	2,482.7	594.9333	14,779,661	3,715.5
2016	505.6767	634.8433	2,943.18	2,588.31	2,406.37	6,564.737	2,211.533	682.3	13,342,802	3,725.8
2015	460.3767	590.9933	2,803.857	2,179.743	2,306	5,796.043	1,975.167	550.2	14,133,201	3,658.2
2014	392.9133	561.24	2,749.31	1,927.66	2,282.877	5,026.42	1,764.2	472.8	14,243,607	3,596.9
2013	326.0267	529.5033	2,571.007	1,702.887	2,150.803	4,283.417	1,565.367	407.9333	12,248,878	3,241.733
2012	259.03	506.7433	2,314.197	1,495.353	1,942.733	3,591.4	1,374.433	333.4667	11,137,943	2,823.967
2011	195.0367	463.69	2,130.183	1,307.067	1,803.953	3,029.2	1,186.833	253.1333	10,489,603	2,374.533
2010	143.34	402.3267	1,707.627	1,040.467	1,428.92	2,924.09	990.3333	189.0667	7,206,392	2,037.733
2009	119.32	366.22	1,306.483	879.0233	1,065.52	221,438	828.1333	151.1667	4,259,594	1,697.567
2008	93.95333	353.46	1,184.937	785.2867	968.9533	1,581.81	714	129.8333	4,539,310	1,388.733
2007	53.2254	301.9233	991.8433	639.65	804.1	1,100.647	572.9667	120.2598	3,149,514	1,120.267
2006	41.01667	262.0467	806.58	538.2167	635.05	894.5233	482.7333	120.4586	2,064,954	1,050.567
2005	62.34667	242.4567	639.1567	470.64	485.1667	725.5333	414.9667	118.2357	1,354,869	917.6333
2004	49.22667	221.5	522.1333	408.6	380	571.0667	353.3	117.5684	1,175,983	782.5667
2003	42.87333	186.6667	401.4433	347.6933	287.77	434.4067	307.7333	115.5368	8,426,867	671.8333
2002	35.2569	178.66	313.9233	324.2433	234.14	296.3467	277.5667	114.6895	5,648,233	568.8667
2001	32.2258	168.6667	262.04	294.52	201.0767	210.6133	254.4333	115.3654	5,103,133	564.3569
2000	30.2635	161.7133	233.5867	272.39	181.2933	172.0267	234.9667	110.02368	541,350	559.2365

Data source: China Statistical Yearbook 2019 and China Tax Yearbook 2019.

### Construction of Jingdezhen ceramic industry taxation fusion model based on support vector machine

**FIGURE 5:**
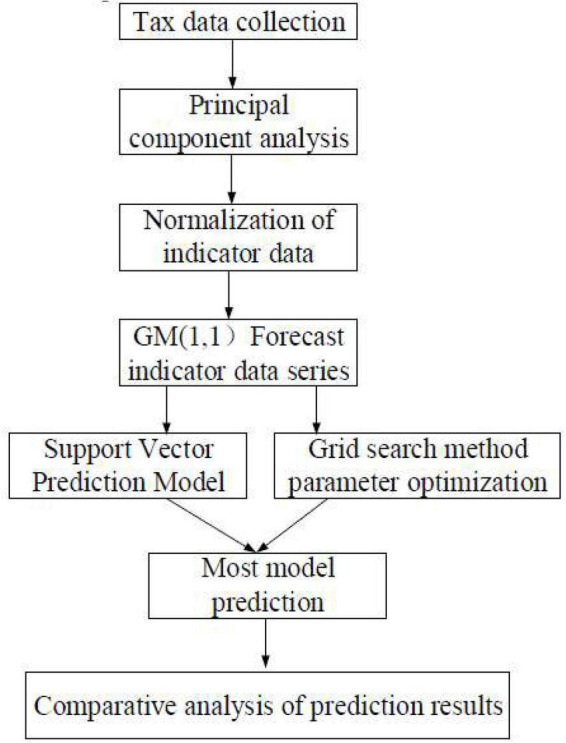
Construction of the taxation fusion model based on support vector machine (SVM) for the ceramic industry in Jingdezhen.

The problems in the tax management of Jingdezhen are analyzed accurately based on the review and research on literature, models, and data. To this end, a sustainable ceramic tax service platform is built, as shown in Figure 5. The specific process is as follows. (1) The major factors affecting tax revenue are determined based on literature and PCA; the tax-related data published by the National Bureau of Statistics are collected according to the indicators. (2) The taxation data are standardized to eliminate the impacts of the dimensional differences between the various indicator factors on the taxation forecast results. (3) The GM (1,1) algorithm is utilized for taxation indicators so that the models are established to solve the forecast values of data in different years. (4) A forecast model is built based on SVM, the model parameters are initialized, and the forecast value of taxation is calculated based on the default value. (5) The training set is learned, the test set is utilized for tests, the taxation forecast results are output, and the errors and effects of the forecast model are analyzed.

## Results

### Principal component analysis of factors affecting the taxation of the ceramic industry

Principal component analysis is carried out on the influential factors of the taxation of the ceramic industry by using mathematical statistical calculation tools. The results in Table 2 indicate that the variance contribution rate of the added value of the primary industry is 0.952; the variance contribution rate of the added value of the secondary industry is 0.0386; the variance contribution rate of the added value of the tertiary industry is 0.0065; the variance contribution rate of the industrial output value is 0.002; the variance contribution rate of fixed asset investment is 0.0004, of which the contribution rate of the first five components reaches 99.95%, and the remaining components only account for 0.05%. Among them, the primary industry refers to the sector that produces products that can be consumed without further processing, such as the agricultural sector. The secondary industry refers to the sector that reprocesses primary products. The tertiary industry refers to the sector that provides various services for production and consumption in the process of reproduction. The most significant tax impact on the ceramic industry is the total output value of the primary industry, followed by the secondary industry. Therefore, the first five metrics are used as principal components and input to the SVM for training and learning.

**TABLE 2:** Principal component analysis (PCA) results of factors affecting the taxation of ceramic industry.

Number	Standard deviation	Variance contribution rate	Cumulative variance contribution rate
X1	2.9271	0.9520	0.9520
X2	0.5895	0.0386	0.9906
X3	0.2429	0.0065	0.9971
X4	0.1368	0.002	0.9991
X5	0.0603	0.0004	0.9995
X6	0.0504	0.0002	0.9997
X7	0.0178	0.0001	0.9998
X8	0.0145	0.0001	0.9999
X9	0.0058	0.0001	1.0000

### Parameter optimization of ceramic industry taxation forecast model

**FIGURE 6:**
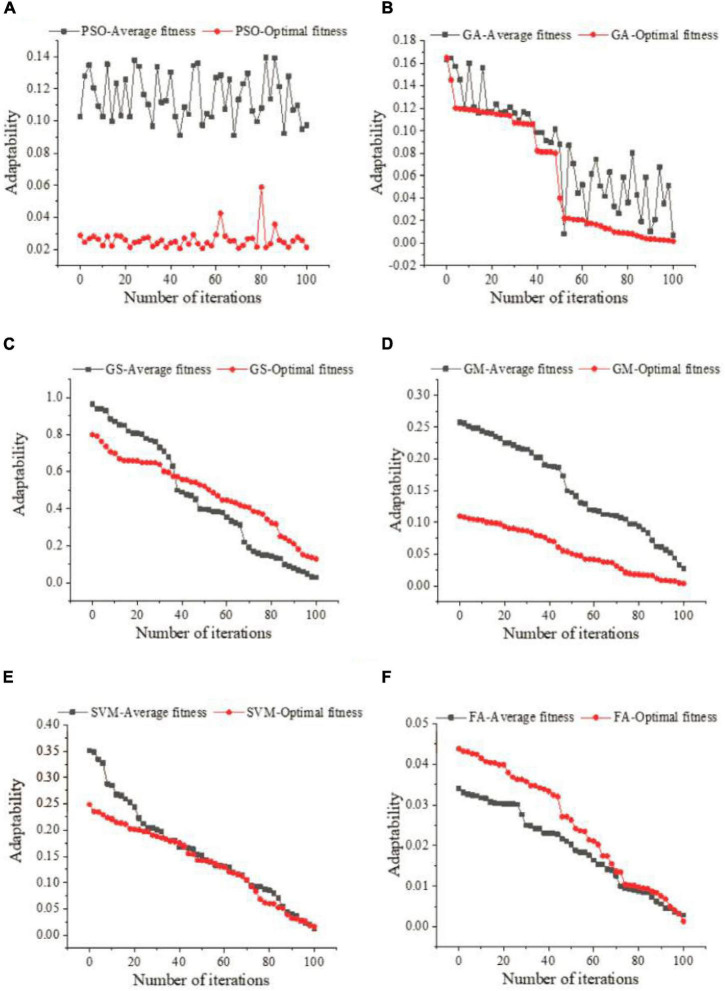
**(A)** Parameter optimization curve of PSO algorithm. **(B)** Parameter optimization curve of GA algorithm. **(C)** Parameter optimization curve of GS algorithm. **(D)** Parameter optimization curve of GM algorithm. **(E)** Parameter optimization curve of SVM algorithm. **(F)** Parameter optimization curve of FA algorithm.

Figure 6 presents the parameter optimization experimental results of the six algorithms. Figure 6 suggests that the overall fluctuation of the PSO algorithm is significant, but it can be kept stable in general. The average fitness function of GA decreases as the number of iterations increases. The fitness of the GS algorithm is the best; the optimal fitness and the average fitness appear earlier. For the GM algorithm, the optimal fitness value does not intersect with the average fitness value. In terms of the SVM algorithm, there are many intersections. For the FA, there is no intersection in the early stage; however, a small amount of intersection occurs later. According to the above results, the forecast curve of the six parameter optimization methods has a high degree of fitness with the actual value, and a good forecast effect is achieved. Among them, FA has the best optimization effect, followed by the GS algorithm and the support vector algorithm.

### Performance analysis of the ceramic industry taxation forecast model

**FIGURE 7:**
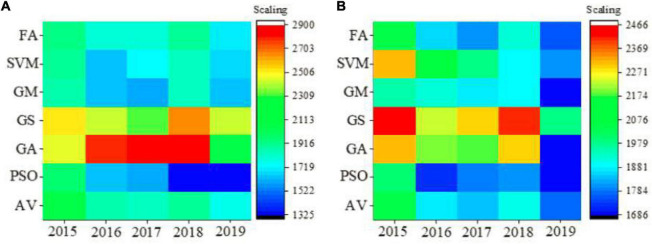
**(A)** Prediction result of tax model of the first industry in ceramic industry. **(B)** Prediction result of tax model of the second industry in ceramic industry.

The two major indicators that affect the principal components of the taxation of the ceramic industry are chosen. The first industry added value and the second industry added value are utilized to evaluate the performance of the model. The results are shown in Figure 7, where actual vector (AV) is the actual value of ceramic tax revenue. According to Figure 7, the FA is superior to other algorithms in terms of the forecast value of the added value of the first industry and the added value of the second industry. In combination with Table 3, it is found that the forecast value of the GM algorithm is less stable, while the results of FA are stable. In addition, the forecast accuracy of FA is also higher than other algorithms. Specifically, the forecast accuracy of FA is improved by 10%, and the absolute error is reduced from 6.9 to 1.8%. Forecasting related industries in Jingdezhen by using the fusion model has found that the primary industry will increase rapidly from 2020 to 2025, while the added value of the secondary industry will increase more slowly; in addition, the tertiary industry will continue to increase, but its added value will increase slowly.

**TABLE 3:** Taxation forecast error results.

Algorithm	MAE	MSE	RMSE
PSO	0.068	−0.04689	0.089
GA	0.055	−0.03584	0.077
GS	0.059	−0.02359	0.069
GM	0.078	−0.01986	0.091
SVM	0.068	−0.06207	0.086
FA	0.018	−0.01191	0.023

### Research on user experience based on human-computer interaction

Five users are invited to evaluate the designed cognitive psychology-based HCI system’s user satisfaction and data analysis accuracy. Table 4 shows the results.

**TABLE 4:** Analysis of product satisfaction based on human-computer interaction (HCI).

Customer number	User satisfaction	Accuracy of data analysis
1	93%	93.4%
2	95.3%	91%
3	95%	93.8%
4	92%	87.5%
5	93.5%	87%

The above results show that users’ satisfaction with the designed HCI system based on cognitive psychology is higher than 92%, which is enough to prove the effectiveness of this method. In addition, the accuracy of data analysis is also more than 87%. To sum up, the HCI system based on cognitive psychology designed can improve the user experience and improve the accuracy of data analysis.

## Discussion

The material basis of economic development is the primary industry, which has been confirmed again by the above experiments. The added value of the primary industry has the greatest impact on taxation. Jingdezhen is the capital of ceramics, and the ceramics manufacturing industry belongs to the secondary industry. However, as the results above show, Jingdezhen’s secondary industry clearly owes taxes. The model prediction results also suggest that the primary industry in Jingdezhen will continue to grow between 2020 and 2025. On the contrary, the growth of the secondary industry will slow down, resulting in tax imbalances and problems with the industrial structure. The above exploration found that Jingdezhen has a series of small workshop-like management systems, with a large number of ceramic employees, but a small proportion of tax revenue. At the same time, large and medium-sized ceramic enterprises have a high tax burden (Li, 2020). These problems hinder the development of the ceramic industry, which has been reported in many literatures. For example, Wu et al. (2020) studied the early development of Jingdezhen ceramic glaze, chemically revealed how the glaze evolved in different periods in Jingdezhen through the chemical analysis of glaze, and proposed a possible early glaze formula. The research revealed the progress and development of Jingdezhen ceramic technology. Nie (2020) studied the economic function data of machine learning and computer interaction platforms. The parameters were selected through the support vector algorithm, and the problem of fast data flow was effectively solved. Kuo et al. (2019) conducted research on HCI system design and college students’ learning motivation. HCI projects all applied interdisciplinary knowledge and skills in the field of Science, Technology, Engineering, and Mathematics. Research showed that computer systems based on HCI technology can help improve the accuracy of economic forecasts. Similarly, the above results also show that the results fluctuate greatly in different algorithms. Therefore, the proposed tax model can provide good ideas for the development of the ceramic industry in Jingdezhen. According to the experimental results, the following countermeasures are proposed. (1) It is recommended that the Municipal Party Committee and the Municipal Government of Jingdezhen City attach great importance to the current taxation status, set up a leading group, integrate the current resources, and enhance the integrating strength of the enterprises. (2) By strictly implementing the taxation system and adding law enforcement personnel, the unqualified enterprises are cleaned up comprehensively to ensure the market order. (3) A ceramic marketing platform is built to manage the production, sales, promotion, and tax collection of the ceramic industry in a way that allows the participants to share information.

## Conclusion

It is the mainstream of modern cognitive psychology to study the process of psychological cognition with the method of information processing. Therefore, the computer management system based on HCI begins to play a vital role in the field of economic and tax forecasting. This work conducts PCA on the factors affecting the taxation of Jingdezhen City and uses different algorithm models to train the taxation data of the ceramic industry in different years in Jingdezhen City. A suitable prediction model is determined through comparison and parameter optimization. In addition, a HCI system is designed based on the cognitive psychology theory, and the impact of Jingdezhen’s tax management on the economic development of the ceramic industry is analyzed. Finally, a reasonable solution is put forward for the taxation problem in Jingdezhen City. The most significant tax impact on the ceramic industry is the total output value of the primary industry, followed by the secondary industry. The tax collection and management fusion model based on SVM has high stability and accuracy. Tax forecasts suggest that Jingdezhen’s tax imbalance has increased. Therefore, the government needs to attach great importance to the development of the ceramic industry, strictly implement tax policies, and implement intensive management. The future tax is predicted and judged based on historical tax data through the SVM tax collection and management fusion model.

Defects still exist due to limited time. Future research should focus on the following aspects. (1) Data should be more diverse. The data structure is relatively simple. For the subsequent prediction results, only the primary and secondary industries are analyzed, and the impact of other indicators is not deeply analyzed. (2) There is no in-depth analysis of the prediction function of the model. Different algorithms are compared and analyzed only through simple prediction analysis. These are worth noting by the future work.

## Data availability statement

The raw data supporting the conclusions of this article will be made available by the author, without undue reservation.

## Ethics statement

The studies involving human participants were reviewed and approved by Jingdezhen Ceramic University Ethics Committee. The patients/participants provided their written informed consent to participate in this study. Written informed consent was obtained from the individual(s) for the publication of any potentially identifiable images or data included in this article.

## Author contributions

The author confirms being the sole contributor of this work and has approved it for publication.
